# Efficient and large-area all vacuum-deposited perovskite light-emitting diodes via spatial confinement

**DOI:** 10.1038/s41467-021-25093-6

**Published:** 2021-08-06

**Authors:** Peipei Du, Jinghui Li, Liang Wang, Liang Sun, Xi Wang, Xiang Xu, Longbo Yang, Jincong Pang, Wenxi Liang, Jiajun Luo, Ying Ma, Jiang Tang

**Affiliations:** 1 Wuhan National Laboratory for Optoelectronics (WNLO) and School of Optical and Electronic Information, Huazhong University of Science and Technology (HUST), Wuhan, Hubei China; 2 State Key Laboratory of Materials Processing and Die & Mould Technology, School of Materials Science and Engineering, Huazhong University of Science and Technology (HUST), Wuhan, Hubei China

**Keywords:** Lasers, LEDs and light sources, Lasers, LEDs and light sources

## Abstract

With rapid advances of perovskite light-emitting diodes (PeLEDs), the large-scale fabrication of patterned PeLEDs towards display panels is of increasing importance. However, most state-of-the-art PeLEDs are fabricated by solution-processed techniques, which are difficult to simultaneously achieve high-resolution pixels and large-scale production. To this end, we construct efficient CsPbBr_3_ PeLEDs employing a vacuum deposition technique, which has been demonstrated as the most successful route for commercial organic LED displays. By carefully controlling the strength of the spatial confinement in CsPbBr_3_ film, its radiative recombination is greatly enhanced while the nonradiative recombination is suppressed. As a result, the external quantum efficiency (EQE) of thermally evaporated PeLED reaches 8.0%, a record for vacuum processed PeLEDs. Benefitting from the excellent uniformity and scalability of the thermal evaporation, we demonstrate PeLED with a functional area up to 40.2 cm^2^ and a peak EQE of 7.1%, representing one of the most efficient large-area PeLEDs. We further achieve high-resolution patterned perovskite film with 100 μm pixels using fine metal masks, laying the foundation for potential display applications. We believe the strategy of confinement strength regulation in thermally evaporated perovskites provides an effective way to process high-efficiency and large-area PeLEDs towards commercial display panels.

## Abstract (web summary)

Overcoming the limitations to fabricate large-area perovskite light-emitting diodes is crucial for bringing the technology a step closer to commercialization. Here, the authors report a 40.2 cm^2^ large-area device with an EQE of 7.1% by using the thermal evaporation method.

## Introduction

The ubiquity of display technology in our day-to-day lives brings a trillion-dollar market worldwide, great efforts thus have been directed towards the development of high-performance and cost-effective display technologies. The recently emerged perovskite light-emitting diodes (PeLEDs) have attracted numerous interests in both academic and industrial fields, due to their high efficiency, narrow emission linewidth as well as low costs of material and fabrication^1–5^. Specifically, the CIE coordinates of PeLEDs could reach 140% of the NTSC standard^6^, which is much wider than those of industrially dominant liquid crystal displays (LCDs) and organic light-emitting diodes (OLEDs) technologies, and the raw material costs of PeLEDs are much cheaper than those of OLEDs. Since the first report of PeLEDs in 2014 by Tan and co-workers^7^, substantial progress has been made in the past several years, achieving an external quantum efficiency (EQE) greater than 20%, which is comparable with that of the best-performing OLEDs^8–10^. These breakthroughs represent an encouraging step towards the practical application of PeLEDs for next-generation display technologies.

For a full-color display, blue, green, and red-emitting pixels with well-designed size and shape are periodically patterned, which are driven by the thin-film transistors (TFT) underneath. The manufacturing of display panels thus relies critically on the integration of large-scale perovskite micro-arrays and TFT panels. Currently, most PeLEDs are fabricated by solution-processed methods with the best performance achieved in functional areas below 0.1 cm^2^, ^11^. One impressive exception is reported by Wang et al. who demonstrated a high EQE of over 16% for their spin-coated PeLED employing a molecular modification strategy^12^. A further scale-up using blade-coating enables an ultra-large PeLED with an area of 4 cm × 7 cm; however, no efficiency was reported for this PeLED^13^. It should also be noted that both spin-coating^14^ and blade-coating are difficult to integrate red, green, and blue perovskite pixels on the pre-pixelated TFT panels with high resolution. Inkjet printing patterned PeLEDs may offer one possible solution, however, they show inferior device performance with a maximum EQE of 3% due to nonideal film quality and “coffee ring” effect^15,16^. Thereby, the state-of-the-art solution-processed PeLEDs remain a significant gap towards full-color display applications. It is thus of urgent importance to develop rational strategies to manufacture efficient and large-area patterned PeLEDs for next-generation display technologies.

Thermal evaporation has been widely used in the semiconductor industry due to its remarkable scalability and reproducibility, as successfully demonstrated by commercial OLEDs^17,18^. Side-by-side three-color perovskite patterns can be easily achieved through sequent deposition with the assistance of fine metal masks, which resemble that of OLEDs. It can be expected that the mature evaporation process and facilities can promote the fabrication of PeLED display panels (Supplementary Table 1)^19,20^. Notably, thermal evaporation is free of toxic solvent and particularly advantageous for depositing hardly soluble inorganic perovskites, such as CsPbX_3_ (X = Cl, Br, I)^21^. Besides, thermal evaporation has been successfully demonstrated by perovskite photovoltaics with power conversion efficiency beyond 20%, suggesting high film quality via thermal evaporation^22^.

Despite holding great promises, the explorations on thermally evaporated PeLEDs are very limited, and their device performance lags significantly behind, which calls for further improvements^11^. In thermally evaporated CsPbBr_3_ films, the three dimensional (3D) network of corner-sharing [PbX_6_]^4−^ octahedra enables large band dispersion and small exciton binding energy at room temperature, indicating that the excitons readily dissociate into free carriers, which are easily captured by nonradiative recombination centers and result in poor photoluminescence^23,24^. Previous studies have demonstrated the most efficient way to solve this problem is by designing low-dimensional or multiple quantum well structures in perovskite emitters, aiming to enhance radiative recombination rate by strongly confining the electrons and holes^25–27^. However, a fundamental study of the spatial confinement, the associated recombination dynamics, and device behavior in the Cs-Pb-Br system, which can provide useful guidelines for the optimization of thermally evaporated PeLEDs, is still lacking^23,28^.

Herein, we report the development of efficient and large-area PeLEDs based on vacuum-deposited Cs-Pb-Br films via spatial confinement. By incorporating zero-dimensional (0D) structural Cs_4_PbBr_6_ into CsPbBr_3_ film, the 3D connection of [PbBr_6_]^4−^ octahedra is partially broken, forming Cs_4_PbBr_6_/CsPbBr_3_ core-shell structure. The electrons and holes formed in CsPbBr_3_ are spatially confined by Cs_4_PbBr_6_. As expected, a blue shift in photoluminescence (PL) wavelength from 516–480 nm is observed as the CsBr/PbBr_2_ molar ratio increased from 1.24–2.28. We then further investigated their carrier recombination dynamics via femtosecond-transient absorption and found that perovskite film of CsBr/PbBr_2_ = 1.56 shows enhanced radiative recombination rate and suppressed nonradiative recombination rate compared to that of CsBr/PbBr_2_ = 1.24. Based on an optimal molar ratio of CsBr/PbBr_2_, we then constructed the most efficient thermally evaporated PeLED with a record EQE of 8.0%. Moreover, we successfully fabricated large-area (10 cm × 9 cm) and patterned perovskite films (the diameter of pixels is ~100 μm), emphasizing the great superiority of our thermal evaporation process. More importantly, we achieved a 7.1% EQE for the 40.2 cm^2^ device, representing one of the most efficient large-area PeLEDs. We believe our works represent an encouraging stepping stone to bring state-of-the-art PeLEDs from the laboratory towards commercial display panels.

## Results

### Manipulation of spatial confinement in thermally evaporated Cs-Pb-Br film

**Fig. 1:**
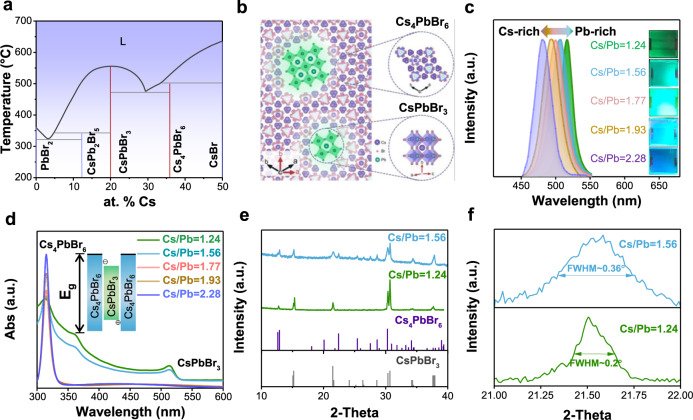
Characterizations of thermally evaporated Cs-Pb-Br film. **a** The phase diagram of CsBr-PbBr_2_ binary system, which is provided under licence from the online database of SpringerMaterials^49, 50^. **b** The schematic diagram of CsPbBr_3_ embedded in the Cs_4_PbBr_6_ matrix, which indicates the 3D [PbBr_6_]^4−^ of CsPbBr_3_ is separated by 0D [PbBr_6_]^4−^ of Cs_4_PbBr_6_. **c**, **d** The PL (**c**) and absorption (**d**) spectra of Cs-Pb-Br films with various Cs/Pb ratios. **e**, **f** XRD (**e**) and its magnification (**f**) for the film of Cs/Pb = 1.56 and Cs/Pb = 1.24.

CsPbBr_3_ has been extensively investigated as a promising material for light-emitting applications, however, the 3D connection of [PbBr_6_]^4−^ gives rise to large band dispersion, enables excitons to dissociate readily, and leads to a slow radiative recombination rate^29,30^. Organic ligands, ammonium halides with large organic cations or polymer matrixes are commonly introduced to enhance radiative recombination rate by providing spatial confinement of electrons and holes^28,31,32^, however, they suffer from low thermal stability due to their organic nature. Figure 1a shows the phase diagram of the CsBr-PbBr_2_ binary system, highlighting not only CsPbBr_3_ but also CsPb_2_Br_5_ and Cs_4_PbBr_6_ are thermodynamically stable phases that can be synthesized based on the different molar ratios of CsBr/PbBr_2_^33^. The Cs-rich phase Cs_4_PbBr_6_ possesses a 0D structure with [PbBr_6_]^4−^ octahedra completely isolated by Cs^+^ cations (Fig. 1b)^34^, which can serve as a barrier to break the connectivity of [PbBr_6_]^4−^ in CsPbBr_3_. Figure 1b shows the crystal model of CsPbBr_3_/Cs_4_PbBr_6_ composites. CsPbBr_3_ can be viewed as nano-inclusions embedded in the Cs_4_PbBr_6_ matrix, which resemble II–VI, IV–VI, and III–V quantum dots and the size of CsPbBr_3_ nano-inclusions determines the strength of quantum confinement effect^35^. Thanks to no intermediate phase between CsPbBr_3_ and Cs_4_PbBr_6_ in the phase diagram (Fig. 1a), the molar ratio of CsBr and PbBr_2_ precursors directly determines the proportions of CsPbBr_3_ and Cs_4_PbBr_6_. Thereby, the strength of the confinement can be controlled by the molar ratio of CsBr/PbBr_2_ instead of introducing foreign organic additives, enabling higher thermal stability compared to organic-inorganic hybrid perovskites (Supplementary Fig. 1). Moreover, the lattice constant of Cs_4_PbBr_6_ matches well with that of CsPbBr_3_, indicating that Cs_4_PbBr_6_ can effectively passivate the surface of CsPbBr_3_ without additional interface strain^35^.

We thus focus on Cs_4_PbBr_6_/CsPbBr_3_ composite films for potential PeLED application. As shown in Fig. 1c, a series of Cs-Pb-Br films with different CsBr/PbBr_2_ molar ratios are fabricated using the thermal co-evaporation (Supplementary Fig. 2), high evaporation rate (Supplementary Fig. 3 and Supplementary Note 1) and low substrate temperature are believed to decrease grain size, further improving spatial confinement (detail in Method section)^36,37^. The CsBr/PbBr_2_ ratio is approximately estimated by an energy-dispersive spectrometer (EDS) measurement (Supplementary Table 2). According to the phase diagram, a high CsBr/PbBr_2_ ratio generally yields high content of Cs_4_PbBr_6_, probably resulting in small-sized CsPbBr_3_ nano-inclusion. The emission color of as-prepared Cs-Pb-Br films varies from green to cyan with the PL peak blue-shifts from 516–480 nm as the ratio of CsBr/PbBr_2_ increases from 1.24–2.28. These results match well with the size-dependent band gap of CsPbBr_3_ nano-inclusions, indicating the enhancement of quantum confinement strength^35^. The absorption spectra also reflect the proportion change of Cs_4_PbBr_6_/CsPbBr_3_ composite films (Fig. 1d). Two absorption peaks located at 315 and 515 nm are attributed to Cs_4_PbBr_6_ and CsPbBr_3_, respectively. As the CsBr/PbBr_2_ precursor ratio increased, the absorption peak from the Cs_4_PbBr_6_ matrix gradually increased while the absorption peak from the CsPbBr_3_ nano-inclusion gradually disappeared, which is consistent with the decreased yellow color in these films (Supplementary Fig. 4). Note that Type-I band alignment of Cs_4_PbBr_6_/CsPbBr_3_ facilitates better confinement of carriers in CsPbBr_3_ with a small bandgap (Fig. 1d, inset)^38^. Due to the moisture instability of Cs-Pb-Br films with high CsBr/PbBr_2_ ratios (Cs/Pb = 2.28, 1.93, 1.77) (Supplementary Fig. 5), we mainly focus on Cs-Pb-Br films with CsBr/PbBr_2_ ratio of 1.56 and 1.24 (named as Cs/Pb = 1.56 and Cs/Pb = 1.24) for further characterization. As shown in Fig. 1e, X-ray diffraction (XRD) patterns reveal the coexistence of CsPbBr_3_ and Cs_4_PbBr_6_. Not surprisingly, the Cs/Pb = 1.56 sample exhibits a stronger Cs_4_PbBr_6_ diffraction peak compared to that of Cs/Pb = 1.24, due to the larger presence of Cs_4_PbBr_6_ within the film. To provide direct evidence for the size change of CsPbBr_3_ nano-inclusion, we magnify the 21.5° peak, which corresponds to the (110) diffraction peak of CsPbBr_3_ (Fig. 1f). According to the Debye-Scherrer formula (*D* *=* *Kλ/Bcosθ*, where *B* is the FWHM and *D* is the mean diameter of the crystal domains)^32^, the relatively broader full width at half maximum (FWHM) in the Cs/Pb = 1.56 sample indicates the reduced size of CsPbBr_3_ nano-inclusions, which is consistent with optical characterizations.

### The effect of spatial confinement on the carrier recombination dynamics

Carrier recombination dynamics play a decisive role in emission behavior. We firstly investigate the excitation-density-dependent PL intensity for determining the carrier recombination types. The initial PL intensity (*I*_*PL*_) shows a quadratic dependence of excited carrier density for the films of Cs/Pb = 1.56 and Cs/Pb = 1.24 (Supplementary Fig. 6), which indicates the radiative recombination process is bimolecular. Meanwhile, by fitting the Arrhenius equation, the exciton binding energies of Cs/Pb = 1.56 and Cs/Pb = 1.24 are derived to be 48.7 and 41.6 meV, respectively (Supplementary Fig. 7)^24,37^. The relatively small exciton binding energies close to the room-temperature thermal energy indicate the dominant recombination mechanism is free-carriers^39^. Thereby, the radiative efficiency $\eta$, i.e. photoluminescence quantum yield (PLQY), could be expressed by the following Eq. 1^23,28^:

1 $$\eta =\frac{{k}_{2}{n}^{2}}{{k}_{1}{{{{{\rm{n}}}}}}+{k}_{2}{n}^{2}+{k}_{3}{n}^{3}}$$

where *n* is the carrier density; *k*_*1*_ is the mono-molecular recombination constant, representing trap-assisted recombination; *k*_*2*_ is the bi-molecular recombination constant, representing radiative recombination; *k*_*3*_ is the tri-molecular recombination constant, representing the nonradiative Auger recombination. As shown in Eq. 1, the radiative efficiency $\eta$ is dependent on the three recombination constants (*k*_*1*_, *k*_*2*_, *k*_*3*_) and carrier density (*n*). In the case of extremely low carrier density, trap-assisted recombination dominates and largely determines the PLQY. As the carrier density increases, the radiative recombination starts to compete over trap-assisted recombination and gradually dominates, thus enhancing PLQY. Under the situation of extremely high carrier density, Auger recombination becomes dominative and decreases PLQY^23^. It is comprehensible that enhancing the radiative recombination rate (larger *k*_*2*_) and suppressing the nonradiative recombination rate (smaller *k*_*1*_ and *k*_*3*_) could guarantee a higher emissive efficiency.

To quantitatively analyze the recombination dynamics of thermally evaporated Cs-Pb-Br films with different confinement strength, we firstly investigate the *k*_*1*_ through time-resolved photoluminescence (TRPL) under extremely low excitation power (Supplementary Fig. 8) where the trap-assisted recombination dominates and high-order recombination contribution is negligible^30^. Consequently, the film of Cs/Pb = 1.56 (*k*_*1*_: 7.14 × 10^8^ s^−1^) possesses a lower trap-assisted nonradiative recombination rate than Cs/Pb = 1.24 (*k*_*1*_: 1.69 × 10^9^ s^−1^), attributable to the better interface passivation as more Cs_4_PbBr_6_ presented in the Cs/Pb = 1.56 sample^40^. To strengthen our conclusion, we directly characterize the defect densities using space-charge limited current (SCLC) measurement with a device structure of ITO/PEDOT/perovskite/TAPC/MoO_3_/Al (Supplementary Fig. 9)^41^. As expected, the Cs/Pb = 1.56 shows a relatively lower defect density of 6.22 × 10^15^ cm^−3^ compared to Cs/Pb = 1.24 (7.83 × 10^15^ cm^−3^)^42,43^.

Moreover, the charge-carrier recombination kinetics can be quantitatively described by the following Eq. 2:

2 $$-\frac{dn(t)}{dt}={k}_{1}\cdot n(t)+{k}_{2}\cdot n{(t)}^{2}+{k}_{3}\cdot n{(t)}^{3}$$

**Fig. 2:**
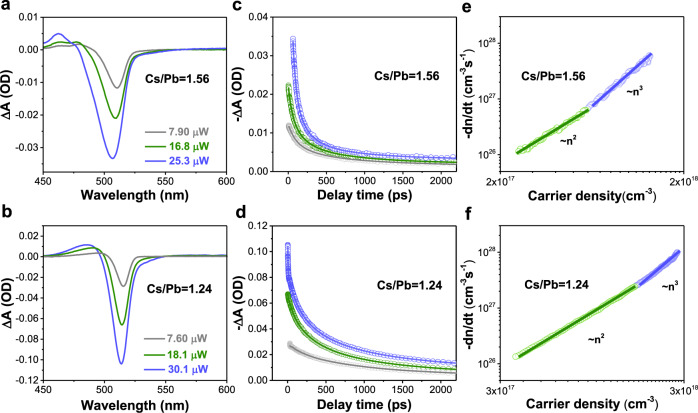
The recombination dynamics of thermally evaporated Cs-Pb-Br films. **a**, **b** The transient absorption spectra of Cs/Pb = 1.56 (**a**) and Cs/Pb = 1.24 (**b**) under 1 ps delay time with various excitation densities. **c**, **d** The corresponding dynamic curves of Cs/Pb = 1.56 (**c**) and Cs/Pb = 1.24 (**d**). **e**, **f** The relationship between carrier recombination decay rates and carrier density in the cases of Cs/Pb = 1.56 (**e**) and Cs/Pb = 1.24 (**f**). The detailed fitting method is described in the Supplementary Information.

The *k*_*2*_ and *k*_*3*_ can be extracted by fitting transient absorption (TA) spectra under various excitation fluences with Eq. 2 (Fig. 2a–f)^28,44^. The fitting results are summarized in Table 1 (calculation details in Supplementary Information and Supplementary Fig. 10). The bi-molecular recombination constant *k*_*2*_ of Cs/Pb = 1.56 is about 8 times higher than that of Cs/Pb = 1.24, confirming enhanced radiative recombination rate due to stronger carrier confinement in the sample with higher Cs_4_PbBr_6_ content. As expected, the measured PLQY of Cs/Pb = 1.56 (40.8%) is indeed higher than that of the Cs/Pb = 1.24 (21.5%) under the moderate excitation fluence. We note that the Auger recombination constant *k*_*3*_ of Cs/Pb = 1.56 is also about 6 times higher than that of Cs/Pb = 1.24, which are attributed to the smaller CsPbBr_3_ nano-inclusions and stronger confinement^28^. In conclusion, the incorporation of the Cs_4_PbBr_6_ phase can not only reduce the trap-assisted recombination rate *k*_*1*_ but also significantly increase the bi-molecular recombination rate *k*_*2*_, which is promising to improve PeLED performance under working conditions (i.e., the electrically injected charge-carrier density is typically lower than that Auger recombination dominates).

**Table 1:** The fitted results of recombination rate constants for thermally evaporated perovskite films with different Cs_4_PbBr_6_ concentrations.

Materials	*k*_*1*_ (s^−1^)	*k*_*2*_ (cm^3^s^−1^)	*k*_*3*_ (cm^6^s^−1^)
Cs/Pb = 1.56	7.14 × 10^8^	9.34 × 10^−10^	1.83 × 10^−27^
Cs/Pb = 1.24	1.69 × 10^9^	1.47 × 10^−10^	3.16 × 10^−28^

The *k*_*1*_ is extracted from time-resolved PL under the low excitation fluence, and *k*_*2*_, *k*_*3*_ are obtained from the global fitting of recombination rates-carrier density curves.

### The relationship between carrier recombination dynamics and device performance

**Fig. 3:**
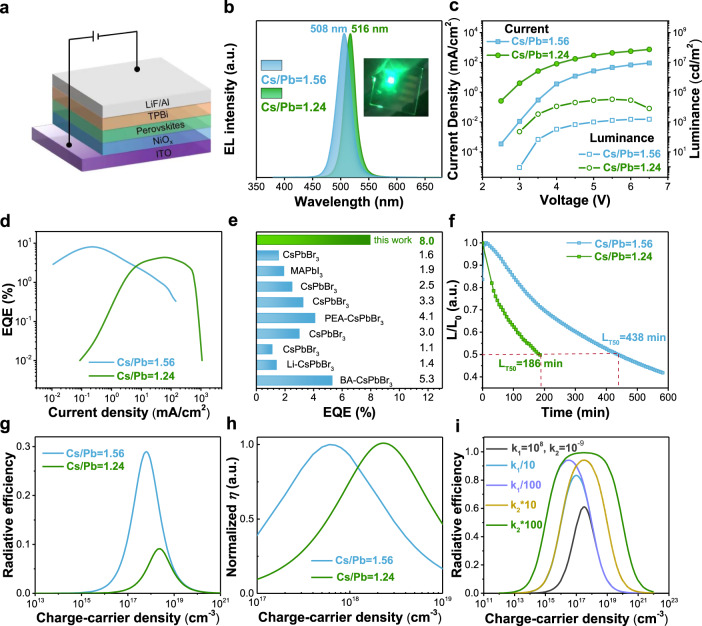
The performance of Cs-Pb-Br PeLEDs. **a** The diagram of PeLED structure. **b** The normalized electroluminescence spectra of Cs/Pb = 1.56 and Cs/Pb = 1.24, the inset is the photo of a Cs/Pb = 1.24 LED driven at 4 V. **c** Current density and luminance versus voltage characteristics of the PeLEDs. **d** EQE-current density curves of these two devices. **e** The EQE summary of thermally evaporated PeLEDs so far reported^25–27, 36, 51–55^. **f** Operational stability of devices at an initial luminance of 100 cd/m^2^. **g** The simulated radiative efficiency evolution with carrier density by using the fitted recombination constants. **h** The normalized radiative efficiency *η* as a function of the carrier density. **i** The effect of *k*_*1*_ and *k*_*2*_ on the radiative efficiency with *k*_*3*_ fixed. k_1_/10 means *k*_*1*_ is 10^7^ while *k*_*2*_ remains invariable (10^−9^).

Encouraged by the enhanced *k*_*2*_ and suppressed *k*_*1*_ through Cs_4_PbBr_6_ incorporation, we construct thermally evaporated Cs-Pb-Br PeLEDs with a device architecture of indium tin oxide (ITO)/Li doped NiO_*x*_/thermally evaporated perovskites/1,3,5-tris(1-phenyl-1H-benzimidazol-2-yl)benzene (TPBi)/LiF/Al. Figure 3a shows the diagram of our PeLED, where the Li-doped NiO_*x*_ serves as a hole transport layer. Note that Li-doped NiO_*x*_ is sputtered onto ITO substrate at room temperature to enhance the hole injection of PeLEDs (Supplementary Fig. 11). The uniformity and compactness of our NiO_*x*_ layer are characterized by the conductive atomic force microscope (c-AFM), where the conductance mapping shows even-distributed current without significant leakage sites (Supplementary Fig. 12). Figure 3b shows the EL spectra of thermally evaporated PeLEDs, with characteristic green emission at 508 and 516 nm, respectively. The blue shift of EL peak for the film of Cs/Pb = 1.56 should result from the confinement effect, consistent with the PL spectra. The EL peak positions of the PeLEDs remain constant with increasing applied voltages up to 6.0 V, suggesting the superior color stability of our PeLEDs (Supplementary Fig. 13). Figure 3c plots the current density-voltage-luminance curves of our thermally evaporated PeLEDs, and the current density of PeLEDs with Cs/Pb = 1.56 is lower than that of PeLEDs with Cs/Pb = 1.24, which is attributed to the reduced carrier mobility with higher Cs_4_PbBr_6_ content (Supplementary Fig. 9)^45^. As expected, PeLED with Cs/Pb = 1.56 demonstrates improved device performance of 8.0% EQE, exceeding that of all previously reported thermally evaporated PeLEDs (Fig. 3e). Supplementary Fig. 14 presents the histogram of the maximum EQE values for 30 devices, which show an average EQE of 6.8% with a low standard deviation (1.1%), demonstrating good reproducibility of our thermally evaporated PeLEDs. It appears that the Cs/Pb = 1.24 sample shows relatively severer EQE roll-off, which may be ascribed to charge imbalance caused by different electron/hole mobility (Supplementary Table 3) and Joule heating generated by the larger current density. Operational stability is another key parameter for PeLEDs. Operated at an initial luminance of 100 cd/m^2^, PeLEDs with Cs/Pb = 1.56 exhibit a superior L_T50_ (indicating the time that the luminance decreased to 50% of the initial value) of 438 min, which is much longer than that of PeLEDs with Cs/Pb = 1.24 (Fig. 3f). Such operational stability is attributed to the improved efficiency and all-inorganic nature of perovskite film, and this represents one of the most stable thermally evaporated PeLEDs and is comparable with that of solution-processed ones^46,47^.

To provide insights concerning the relationship between charge recombination dynamics and device performance, we compare the EQE-current density curves with the radiative efficiency (*η*)-carrier density evolution to investigate the influence of spatial confinement on the LED performance. For the electro-excited PeLED devices, the radiative efficiency (*η*) is positively correlated to the internal quantum efficiency, where radiative efficiency *η* can be calculated based on Eq. 1 using the recombination constants in Table 1. Figure 3g presents the simulated *η* versus excited carrier density (which is associated with current density), indicating higher peak radiative efficiency of PeLEDs with Cs/Pb = 1.56 compared with that of Cs/Pb = 1.24, which is consistent with the behavior of our PeLEDs. For deep understanding, the maximal radiative efficiency (*η*_max_) can be expressed as Eq. 3^30^:

3 $${\eta }_{{\max }}=\frac{1}{1+2\sqrt{{k}_{1}{k}_{3}}/{k}_{2}}$$

From Eq. 3, the *η*_max_ is positively proportional to *k*_*2*_ and negatively proportional to *k*_*1*_, thereby the LED of Cs/Pb = 1.56 with higher *k*_*2*_ and smaller *k*_*1*_ should obtain better EQE. Figure 3h shows the normalized *η* evolution with carrier density, indicating peak *η* can be obtained at a relatively low carrier density with increasing Cs_4_PbBr_6_. This trend is similar to the EQE-current density curves in Fig. 3d.

Despite great progress achieved in EQE improvement of thermally evaporated PeLEDs, a maximum EQE of 8% is still lower than that of solution-processed ones. To further propose possible methods for achieving highly efficient thermally evaporated Cs-Pb-Br LEDs, based on the Cs/Pb = 1.56, we modify the *k*_*1*_ and *k*_*2*_ by one and two orders of magnitude to observe the change of *η*-current density curves. As shown in Fig. 3i, at the same order of magnitude, the influence of *k*_*2*_ on peak radiative efficiency is more prominent. Meanwhile, the shape of *η*-current density curves gradually becomes broader with the increase of *k*_*2*_, which indicates it will maintain moderate efficiency over a wider range of carrier density namely improved roll-off. From this perspective, focusing on increasing *k*_*2*_ may be a more efficient way to boost device performance. Fundamentally, *k*_*2*_ is directly correlated with the wavefunction overlap between electrons and holes, which can be controlled by the spatial confinement effect. For our thermally evaporated CsPbBr_3_, the incorporation of Cs_4_PbBr_6_ breaks the 3D connection of [PbBr_6_]^4−^ to provide spatial confinement of electrons and holes, leading to the significant improvement of *k*_*2*_. However, the encapsulation of CsPbBr_3_ by Cs_4_PbBr_6_ is not fully controllable in our thermally evaporated film. As a result, CsPbBr_3_ domains with partial or completely none Cs_4_PbBr_6_ are nearly inevitable, leading to inadequate enhancement of *k*_*2*_. Nonetheless, a record EQE of 8.0% of our thermally evaporated PeLEDs is very encouraging considering the very limited optimization done so far. We believe further optimization towards > 20% EQE is in near future.

### Demonstration of large-area and patterned PeLEDs

**Fig. 4:**
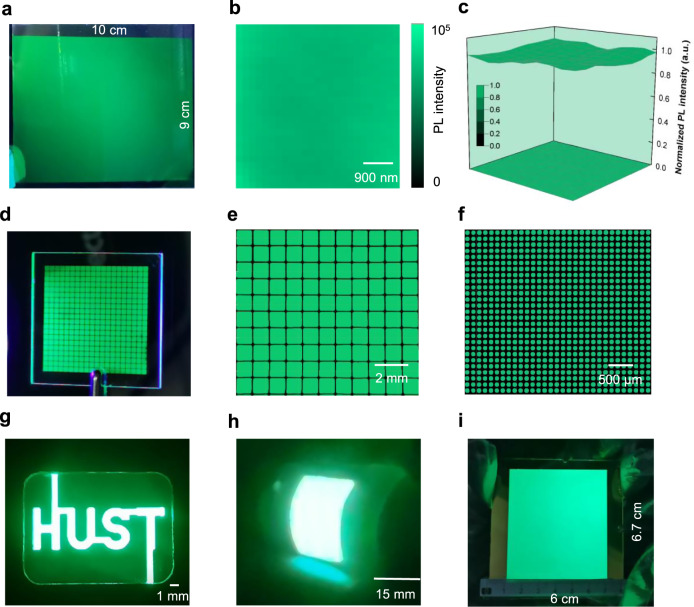
Potential of thermally evaporated PeLED for display application. **a** PL image of 90 cm^2^ Cs-Pb-Br film fabricated by thermal evaporation. **b**,**c** The spatial distribution of PL intensity for the micro area (5 μm × 5 μm) and macro area (90 cm^2^). **d** The PL photo of CsPbBr_3_ film with 900 μm × 900 μm pixels. **e**, **f** The images of fluorescence microscopy characterization for the patterned perovskite film. **g** EL photo of PeLED with a “HUST” pattern (48 mm^2^). **h** A flexible PeLED with an electroluminescent area of 300 mm^2^. **i** Photograph 40.2 cm^2^ PeLED under working conditions.

To further demonstrate the uniformity and scalability of the thermal evaporation technique, large-area Cs-Pb-Br film reaching 90 cm^2^ is achieved for the first time. Figure 4a shows the photograph of as-prepared film, indicating uniform PL emission under ultraviolet light irradiation. Furthermore, we measure the PL mapping to characterize the uniformity of our large-area perovskite films. Encouragingly, the as-prepared film shows uniform PL emission with negligible fluctuations not only on the micro-scale (5 μm × 5 μm) (Fig. 4b) but also on the macroscopic scale (90 cm^2^) (Fig. 4c), demonstrating the superior uniformity of the thermally evaporated film. Besides, the micro-area distribution of PL peak positions and FWHM also show little fluctuations (Supplementary Fig. 15). In contrast, large PL emission fluctuations, especially at edge areas, are generally found for solution-processed films^13^. Moreover, the morphology and composition uniformity of our large-area films are also characterized. SEM images show identical morphology for the center and edge sites (Supplementary Fig. 16). Meanwhile, the element distribution of Cs, Pb, Br is uniform not only on the micro-scale but also for the whole film (Supplementary Fig. 17 and Supplementary Fig. 18). The great uniformity of our thermally evaporated film lays the foundation for large-area and efficient PeLED devices.

Furthermore, we can easily realize perovskite patterns using fine metal masks, which resemble OLED displays. Figure 4d shows the photograph of as-prepared perovskite patterns with a pixel size of 900 μm × 900 μm and pixel diameter of ~100 μm, exhibiting bright and uniform green emission under ultraviolet light irradiation. Fluorescence microscope characterization further reveals uniform PL emission without any visible defect appeared at each pixel (Fig. 4e and Fig. 4f), and the edge area is clear and sharp. In contrast, the ink-printing perovskite patterns often show nonuniform morphology due to the serious “coffee ring” effect^15^. We believe these results demonstrate the superiority of thermally evaporated techniques for the fabrication of perovskite patterns. The maturity of thermal evaporating technologies and facilities will certainly facilitate the preparation of PeLED displays.

**Fig. 5:**
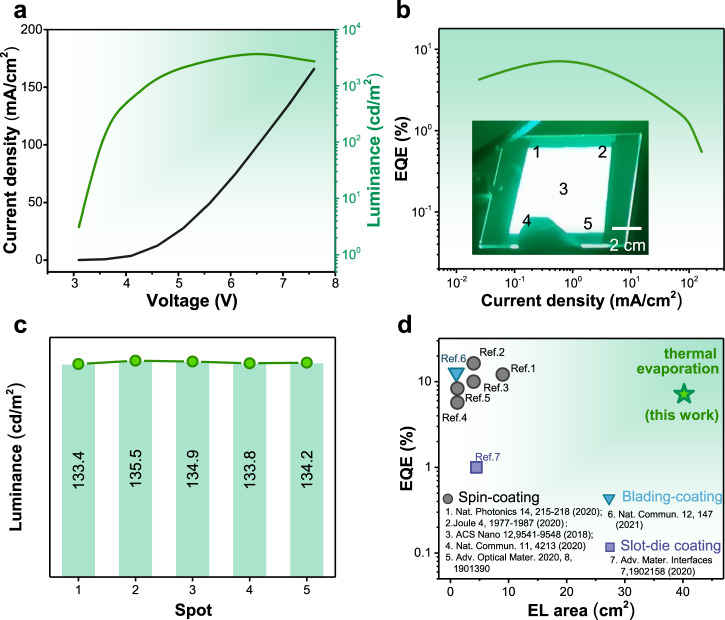
The performance of large-area PeLEDs. **a** Current density-voltage-luminance curves. **b** EQE-current density curve. **c** The luminance of spots 1–5 (corresponding to the marked ones in panel **b**, inset) for our PeLED. **d** The reported EQE of large-area PeLEDs (exceed 1 cm^2^) based on various deposition strategies. Clearly, thermal evaporation shows enormous potential for efficient and large-size PeLEDs.

Encouraged by these findings, we further demonstrate large-area PeLEDs on both rigid glass/ITO substrates and flexible PET/ITO substrates. Figure 4g shows the small-area (48 mm^2^) PeLEDs with a logo of ‘HUST’, demonstrating the patterning of our thermally evaporated PeLEDs. Figure 4h demonstrates a flexible device with good flexibility under working conditions, which shows uniform electroluminescence at a bending angle of about 45°. To further demonstrate the scalability of our thermally evaporated technique, an ultra-large PeLED with a functional area up to 40.2 cm^2^ was fabricated (Fig. 4i), which is the largest one ever achieved in PeLEDs (Supplementary Table 4). We measured the current density-luminance-voltage (*J-V-L*) and EQE-current density (*EQE-J*) curves of our PeLED (Fig. 5a, b). Encouragingly, the as-prepared device delivers a peak EQE of 7.1% with uniform EL emission over the whole working area (Fig. 5c), showing a slight EQE loss of 0.9% when functional areas are scaled up from 0.05–40.2 cm^2^. In comparison, the typical EQE of solution processed-PeLED reduces from 20.2–12.1% when devices scaled up from 0.04–9 cm^2^ ^14^. We believe the well-controlled deposition during the thermal evaporation process and excellent film uniformity over a large area together contribute to the small EQE loss^22^. We noted that such a large area of 40.2 cm^2^ is comparable with the commercial cellphone screen, highlighting their great potential to be built on a commercial display screen, and 40.2 cm^2^ is not the limit for thermally evaporated PeLEDs considering 65-inch OLED display panels have already been mass-produced^48^. Figure 5d summarizes the performance of large-area PeLEDs fabricated based on various film-coating methods. The EQE of 7.1% is one of the highest ever reported for PeLEDs with functional areas exceeding 10 cm^2^. Moreover, our all-vacuum processed strategy also shows excellent compatibility with the current OLED mass production line, potentially bringing the commercialization of PeLED technology one step closer.

## Discussion

Slow radiative recombination rate (*k*_*2*_) is the critical limiting factor faced by three-dimensional perovskite CsPbBr_3_, which leads to inferior radiative efficiency. In principle, *k*_*2*_ is directly correlated with the wavefunction overlap between electrons and holes. Thus, we incorporate zero-dimensional Cs_4_PbBr_6_ into three-dimensional CsPbBr_3_ perovskite for providing spatial confinement of electrons and holes by forming type I heterostructures. As a result, we achieve a bright Cs-Pb-Br film with enhanced *k*_*2*_ and suppressed *k*_*1*_. When implemented as active layers in LEDs, they showed an impressive EQE of 8.0%, higher than any previously reported thermally evaporated PeLEDs. Taking advantage of the uniformity and scalability of the thermal evaporation technique, we achieve the largest PeLEDs with an EQE of 7.1% and a functional area up to 40.2 cm^2^, comparable with commercial cellphone screens. Moreover, the maturity of the thermal evaporation technique facilitates the preparation of perovskite patterns and flexible PeLEDs. Our results demonstrate the great potential of thermally evaporated PeLEDs for next-generation display technologies. Further study should continue to improve devices performance by designing better encapsulation of CsPbBr_3_, passivating defects to suppress *k*_*1*_, balancing the high PL and efficient carrier injection, and further improving device stability.

## Methods

### Materials and chemicals

CsBr (99.999%) and PbBr_2_ (99.999%) were purchased from Sigma-Aldrich. LiF was purchased from Aladdin Reagent Ltd. TPBi was purchased from Xi’an Polymer Light Technology Corp. Li-doped NiO_x_ target (NiO: Li_2_O = 98.96:1.04 wt%) and Al pellet (99.999%) were purchased from ZhongNuo Advanced Material Technology Co., Ltd. All chemicals were used as received.

### Perovskite film deposition

The Cs-Pb-Br perovskite films with various Cs/Pb ratios were fabricated by co-evaporation of CsBr and PbBr_2_ in separate crucibles, and we noted that Cs/Pb ratios will affect the morphology of Cs-Pb-Br films (details in the Supplementary Note 2). It should be emphasized that the thickness of CsBr and PbBr_2_ should be calibrated to obtain the correct scale factor; vacuum higher than 1 × 10^−4^ Pa and clean environment of the evaporation chamber is crucial to avoiding pollution of perovskite film, enabling well-performing devices. The films with various Cs/Pb ratios are deposited through the evaporation rate adjustment of CsBr (1.2–3 Å/s) with a fixed rate of PbBr_2_ (1 Å/s) without substrate heating. It is necessary to finely optimize the evaporation rate ratio of Cs/Pb. According to the previous reports, the high evaporation rate will facilitate the formation of immobile nuclei^37^, the low temperature may impede the growth of nuclei^36^, and thereby the combinatorial process is beneficial to produce small grains providing boundaries for the spatial confinement. To accurately control the rate, the shutter is open only when both evaporation rates are stable, and the supply powers are kept constant during the whole deposition to minimize fluctuation.

### Perovskite LED fabrication

The patterned ITO-coated glass substrates were subsequently cleaned using detergent, acetone, deionized water, and isopropyl alcohol by sonication. Then the ITO substrates were transferred into the sputtering system (JCP500, Beijing Techno Technology Co., Ltd) for depositing Li-doped NiO_x_ (NiO: Li_2_O = 98.96:1.04 wt%) hole transport layer by magnetron sputtering. The radiofrequency (RF) sputtering power was set at 200 W without substrate heating, and the flow rate of argon gas was kept at 100 sccm. The thickness of NiO_x_ is about 20 nm. After that, NiO_x_ substrates were transferred into the evaporation chamber for perovskite deposition (80 nm). Our thermal evaporator (R27-E, Shenyang Qihui Vacuum Technology Co., Ltd) is connected with a glove box to minimize ambient exposure during sample transferring and device measurements. Finally, TPBi (17 nm) and LiF/Al electrodes (1 nm/100 nm) were deposited by thermal evaporation under a high vacuum of < 5 × 10^–4^ Pa. The LED emitting area was 0.05 cm^2^ as defined by the overlapping area of ITO and Al electrodes. All of the layers were deposited at room temperature. The 40.2 cm^2^ large-area LED was fabricated with an Al area of 6.7 cm × 6 cm. The “HUST” LED with a total emitting area of 48 mm^2^ was achieved by the patterned Al cathode. As for the flexible LEDs, the polyethylene glycol terephthalate (PET) substrates were used for the bending exhibition, while other functional layers are the same as the conventional rigid devices.

### Perovskite film and device characterizations

The steady-state PL spectra were recorded in the glovebox using a Photo Research Spectra Scan PR655 under the irradiation of the 365 nm UV lamp. The ultraviolet-visible absorption spectra were performed on a SolidSpec-3700 UV/374 vis/near-IR spectrophotometer with an integrating sphere. The XRD patterns were recorded with a Philips X’Pert Pro diffractometer with Cu K α radiation (λ = 1.54 Å). For transient absorption (TA) measurements, femtosecond laser pulses of an 800 nm fundamental beam (5 kHz repetition rate, 35 fs pulse width) were produced using a regenerative amplified Ti: Sapphire (Legend Duo, Coherent Inc.). Part of the fundamental beam was used to pump an optical parametric amplifier (TOPAS-Prime, Light Conversion) to serve as a narrowband pump, while the other part was focused on a sapphire crystal to generate a white-light supercontinuum probe (420–750 nm window with various optical filters). The pump wavelength was 365 nm. Both the pump and probe pulses were directed into a commercial TA spectrometer (Helios, Ultrafast Systems). Delaying the probe pulse relative to the pump provides a time window of up to 8 ns. The instrument response function (IRF) was determined to be ~200 fs by a routine cross-correlation procedure. The transient PL was collected on an Edinburgh Instruments Ltd EPL-370. The spatially resolved PL and temperature-dependent PL were determined using a confocal microscope spectrometer (Alpha300 Raman, WITec) with a 355 nm laser: the peak intensity maps of large-area films were extracted from the PL spectra of each spot sequentially measured by moving film; the PL position, FWHM, and intensity of micro-area mapping were conducted by automatic stepping machine with a 200 nm step. The PL images of patterned perovskite film were collected by confocal fluorescence microscopy. The trap densities of the perovskite films were extracted by the dark current-voltage characteristics of the hole-only devices in the device architecture of ITO/NiO_x_/Perovskite/TAPC/MoO_3_ (10 nm)/Ag (80 nm) through a computer-controlled Keithley 2400 source meter. The EL performances including current density, luminance, EQE, and operational stability (without encapsulation) of the perovskite LEDs were recorded simultaneously by a commercial measurements system (a programmable Keithley 2400 source meter and a Photo Research Spectra Scan PR655) which was integrated with an N_2_ filled glovebox. The thermogravimetric analyses of perovskite crystals were conducted on the Diamond TG/DTA (PerkinElmer Instruments) under an N_2_ atmosphere with a heating rate of 10 °C/min.

## Supplementary information

Supplementary Information

## Data Availability

The data that support the plots within this paper and other findings of this study are available from the corresponding author upon reasonable request. The phase diagram of CsBr-PbBr_2_ is available at: https://materials.springer.com/isp/phase-diagram/docs/c_0202533.
