# Supplementary material for: Efficient and large-area all vacuum-deposited perovskite light-emitting diodes via spatial confinement
Source: Nat Commun. 2021 Aug 6;12:4751. doi: 10.1038/s41467-021-25093-6 (PMC8346511; doi:10.1038/s41467-021-25093-6)
Supplement: Supplementary file 1 — Supplementary Information [file 41467_2021_25093_MOESM1_ESM.pdf]

Supplementary Information

Efficient and large-area all vacuum-deposited perovskite light-emitting diodes via spatial confinement

Peipei Du[1,2†], Jinghui Li[1†], Liang Wang[1†], Liang Sun[1], Xi Wang[1], Xiang Xu[2], Longbo Yang[1], Jincong Pang[1], Wenxi Liang[1], Jiajun Luo[1]*, Ying Ma[2], Jiang Tang[1]*

[1]Wuhan National Laboratory for Optoelectronics (WNLO) and School of Optical and Electronic Information, Huazhong University of Science and Technology (HUST), 1037 Luoyu Road, Wuhan, Hubei 430074, China.
[2]State Key Laboratory of Materials Processing and Die & Mould Technology, School of Materials Science and Engineering, Huazhong University of Science and Technology (HUST), 1037 Luoyu Road, Wuhan, Hubei 430074, China.

* To whom correspondence should be addressed: luojiajun@mail.hust.edu.cn; jtang@mail.hust.edu.cn
† These authors contributed equally to this work.

Supplementary Table 1 The comparison of thermal evaporation method with traditional solution-processed methods for PeLEDs.

Fabrication process	Scalability	Repeatability	Pixelability	Manufacturability	Ref.
Spin-coating	Low	Moderate	Difficult	Low	[1,2]
Inkjet printing	High	Moderate	Easy	Moderate	[3-6]
Blading-coating	High	Moderate	Difficult	Low	[7,8]
**Thermal evaporation**	**High**	**High**	**Easy**	**High**	**This work**

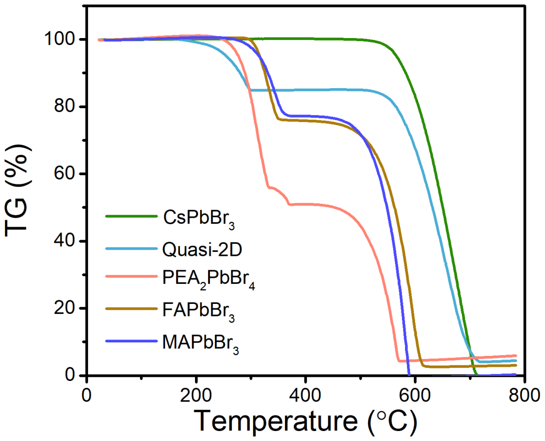

Supplementary Fig. 1 Thermogravimetric analyses of all-inorganic $CsPbBr_3$ and organic-inorganic hybrid perovskites conducted under N_2 atmosphere with a heating rate of 10 °C/min. $CsPbBr_3$ inorganic perovskite begins weight loss at over 500°C.

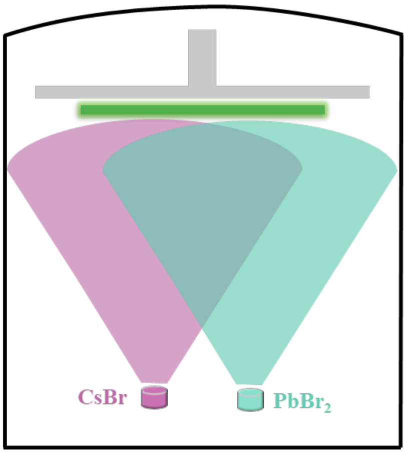

Supplementary Fig. 2 Schematic diagram of co-evaporation for Cs-Pb-Br film deposition. The stoichiometry of the as-deposited film is controlled by adjusting the evaporation rate ratio of CsBr and $PbBr_2$. To further enhance the confinement strength, a high evaporation rate and low substrate temperature (RT) are adopted to expect forming small grain size.

Supplementary Note 1

The evaporation rate is a key parameter in determining the optoelectronic properties of thermally evaporated perovskite films. The growth mechanism of thermally evaporated perovskite follows well with Volmer-Weber growth (Supplementary Fig. 3a), where the initial nucleus will grow to grains and then determine the final grain size. Generally, The more the initial nucleus (larger condensation rate N_d), the smaller final grain size, and the condensation rate N_d is further related to evaporation rate N_e according to the following equation:

$$N_d = \frac{N_e A cos\theta}{4\pi r^2} \alpha_2$$

where A is the area of the source (Supplementary Fig. 3b).

It is clear that evaporation rate N_e will influence the grain size and morphology of our thermally evaporated perovskite films. To gain a better understanding, we have performed additional SEM images of thermally evaporated perovskite films with different evaporation speeds (Supplementary Fig. 3c). The average grain size of the film with a 1 Å/s evaporation rate is relatively smaller than that of 0.5 Å/s, which matches well with the above analysis. Small

grain size is generally beneficial for confining electrons and holes to improve radiative recombination in PeLEDs[9].

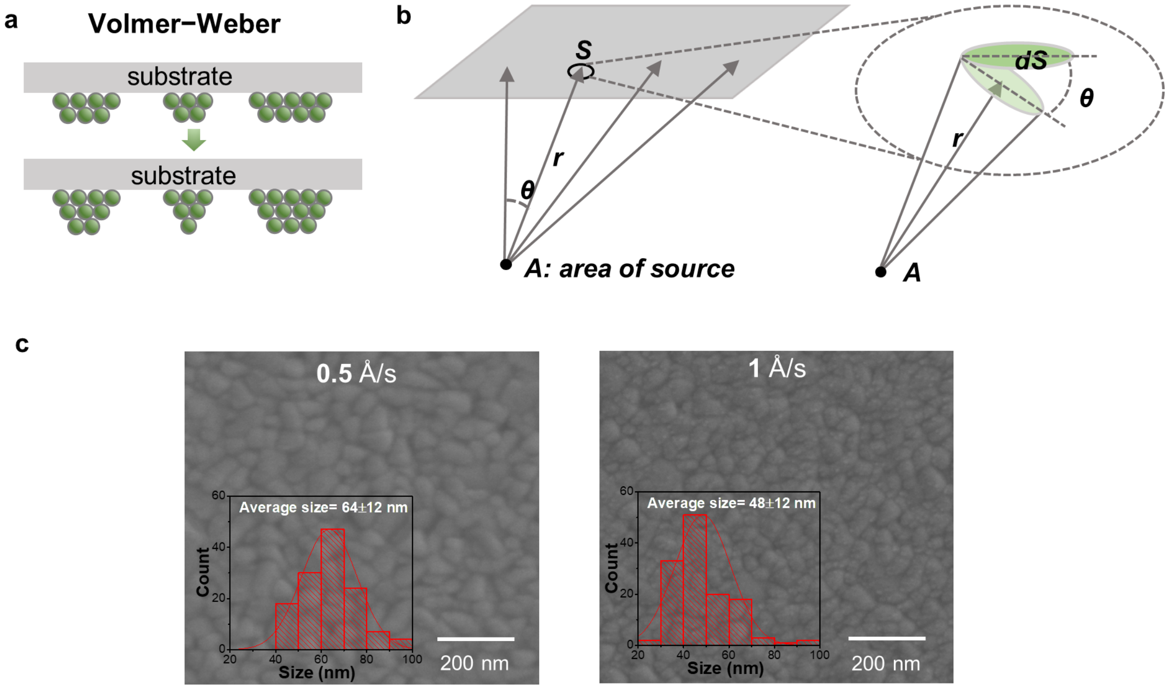

Supplementary Fig. 3 The film growth mechanism. **a** Schematic illustration of the Volmer-Weber growth mechanism. **b** Parameter description in the formula of condensation rate. **c** SEM of films with different evaporation rates.

Supplementary Table 2 Energy-dispersive spectrometer (EDS) results of Cs-Pb-Br films with different Cs/Pb ratios.

PL peak	Cs At%	Pb At%	Cs/Pb ratio
516 nm	17.08	13.72	1.24
508 nm	25.91	16.64	1.56
500 nm	28.19	15.90	1.77
492 nm	26.31	13.63	1.93
480 nm	27.46	12.01	2.28

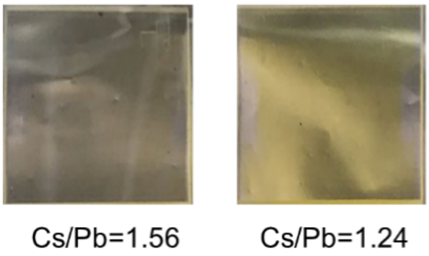

Supplementary Fig. 4 Photographs of Cs/Pb=1.56 and Cs/Pb=1.24. Under daylight, the former is transparent while the latter is semi-transparent, consistent with the absorption character.

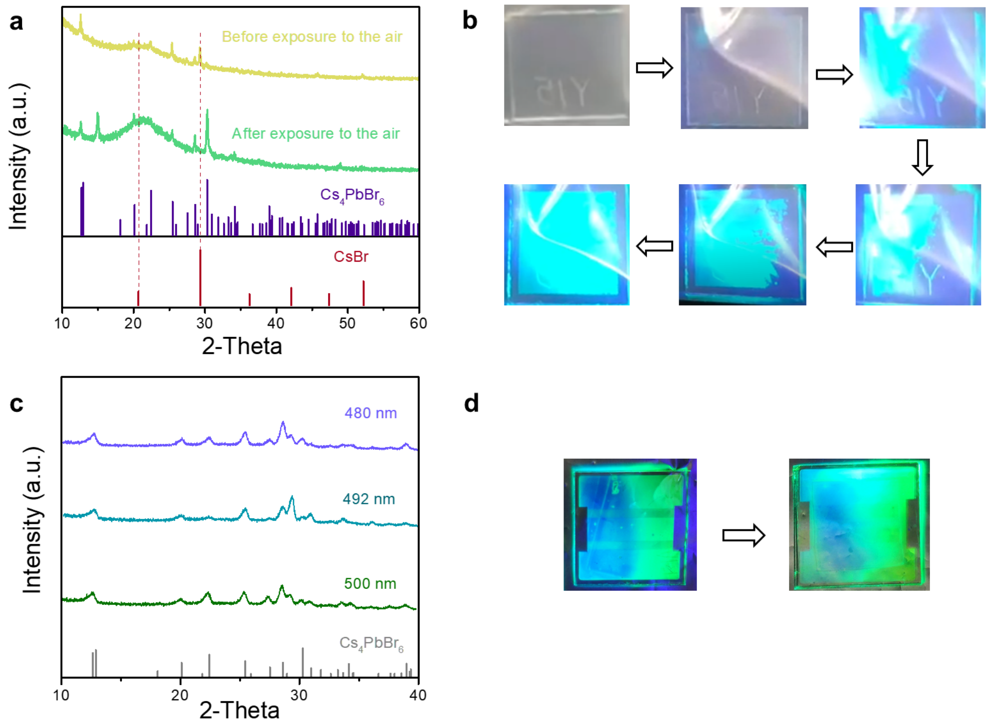

Supplementary Fig. 5 The PL tunability is dependent on the size-induced spatial confinement. **a** Moisture in the air that easily extracts CsBr could trigger the transformation of non-luminescent Cs_4PbBr_6 to PL-active $CsPbBr_3$[10]. **b** The PL evolution of extremely cesium-rich film with the permeation of the external air: at the beginning, the film encapsulated in a nitrogen environment is non-nonluminous (XRD in (a) indicates it is the mixture of Cs_4PbBr_6 and CsBr); after exposure to the air, it emitted bright fluorescence, along with the phase transportation of $Cs_4PbBr_6/CsPbBr_3$. We explain this phenomenon by the reversible reactions: $CsPbBr_3+3CsBr \leftrightharpoons Cs_4PbBr_6$. **c** XRD of Cs-Pb-Br films whose PL peaks correspond to 480 nm, 492 nm, and 500 nm, respectively. It indicates Cs_4PbBr_6 is the dominant phase for these films.

d The blue-fluorescence gradually changed to green-fluorescence after exposure to the air, we attribute to the reduced confinement strength of Cs_4PbBr_6 to $CsPbBr_3$ for moisture decreases the CsBr concentration.

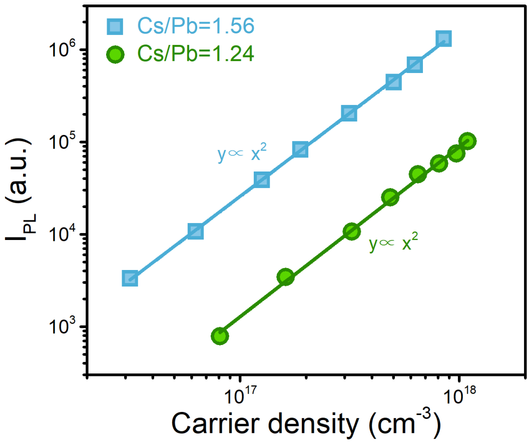

Supplementary Fig. 6 The integrated PL intensity as a function of carrier density. Herein, the carrier density is estimated through the absorption coefficient combined with excitation photon numbers (excitation wavelength： 375 nm).

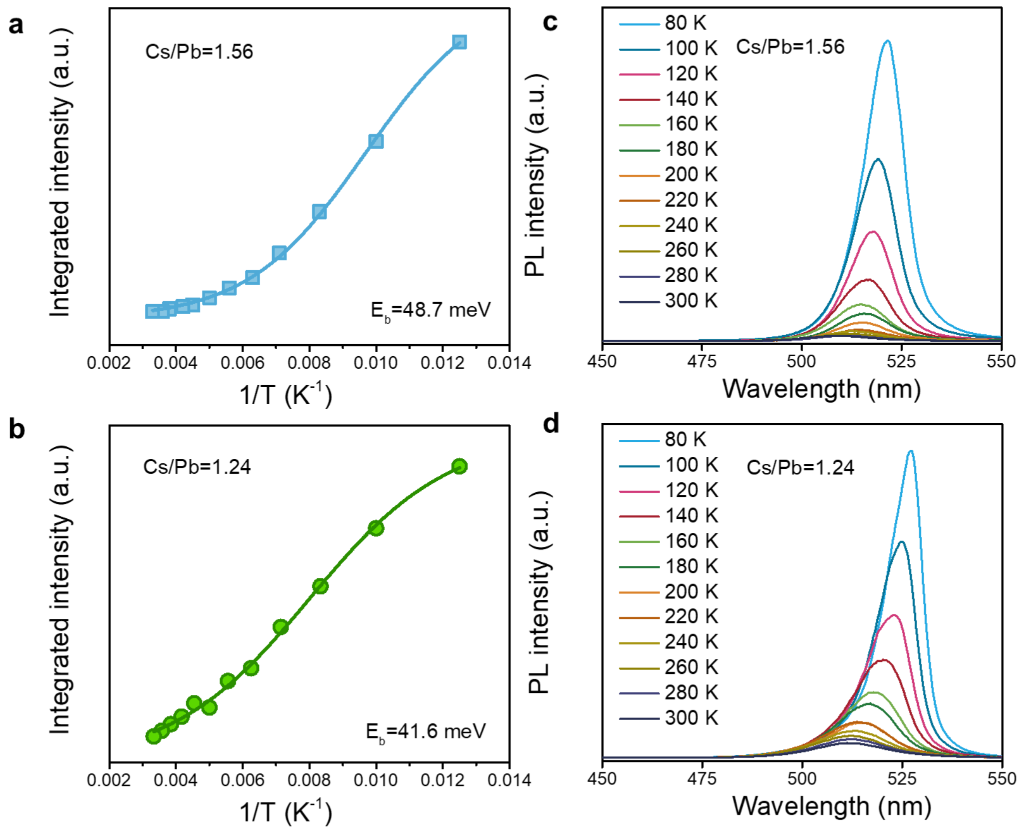

Supplementary Fig. 7 Temperature-dependent PL spectra of Cs/Pb=1.56 and Cs/Pb=1.24. **a**, **b** The integrated PL intensity as a function of temperature for Cs/Pb=1.56 (a) and Cs/Pb=1.24 (b). The PL quenches with the increase of temperature, which always for the thermal dissociation of excitons. Then the fitted activation energy (through equation $I(T)=I_0/(1+A*exp(-E_b/KT))$) corresponds to the exciton binding energy[11]. **c**,**d** PL spectra at different temperatures of the two films.

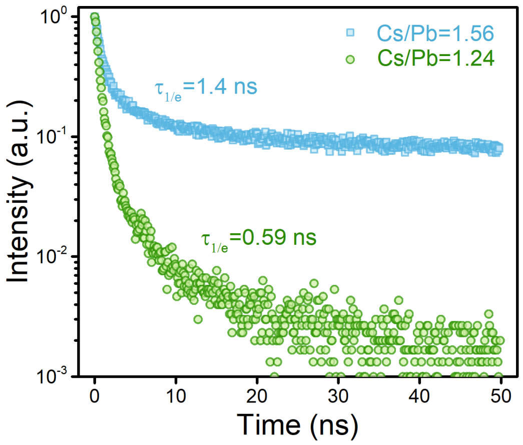

Supplementary Fig. 8 Time-resolved fluorescence spectra of Cs/Pb=1.56 and Cs/Pb=1.24 under extremely low carrier density. The effective lifetime is 1.4 ns and 0.59 ns for Cs/Pb=1.56 and Cs/Pb=1.24. Under low carrier density (~10^{12} cm^{-3}), the bimolecular recombination and Auger recombination are negligible, and the monomolecular recombination constant k_1 can be obtained through the equation ($\tau_{1/e}=1/(k_1+k_2*n/2+k_3*n^2/3)$)[12].

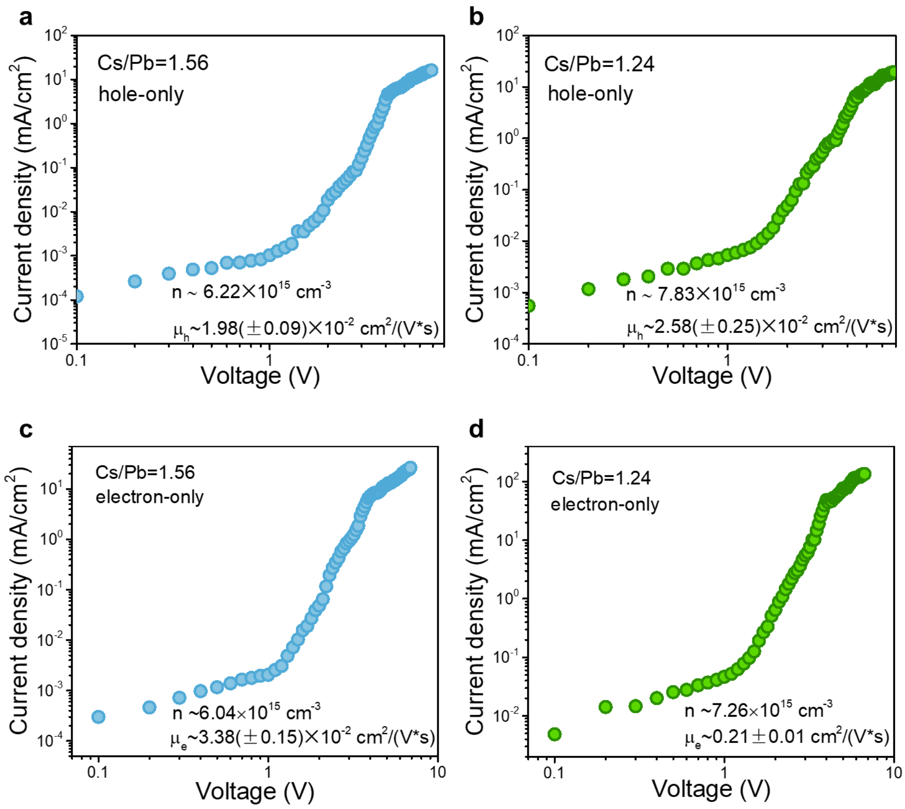

Supplementary Fig. 9 a, b The I-V curves of Cs/Pb=1.56 (a) and Cs/Pb=1.24 (b) devices with structure of ITO/PEDOT/perovskite/TAPC/MoO_3/Al. **c, d** The I-V curves of Cs/Pb=1.56 and Cs/Pb=1.24 devices with structure of ITO/ZnO/perovskite/TPBi/LiF/Al. The defect density is evaluated through the equation of $n=2\varepsilon\cdot V_{TFL}/e\cdot d^2$, where the d (400 nm) is the thickness of perovskite in the whole device and the value of ε for $CsPbBr_3$ was reported as 4.8[13]. The mobility can be extracted from the child region according to the equation of $J=9\varepsilon\mu V^2/8d^3$. The Cs/Pb=1.56 film shows lower defect density and mobility than Cs/Pb=1.24.

Supplementary Table 3 The carrier mobility of Cs-Pb-Br films, which is extracted from Supplementary Fig. 9. The Cs/Pb=1.24 film processes a larger gap between hole mobility and electron mobility causing charge imbalance.

Materials	hole mobility (cm^2/V/s)	electron mobility (cm^2/V/s)	electron/hole
Cs/Pb=1.56	1.98 (±0.09) $\times 10^{-2}$	3.38 (±0.15) $\times 10^{-2}$	~1.7
Cs/Pb=1.24	2.58 (±0.25) $\times 10^{-2}$	0.21±0.01	~8.1

The calculation of carrier recombination constants

In perovskite materials, there are always three pathways of mono-, bi-, and tri-molecular recombination, which can be described as following equation 2:

$$-\frac{dn(t)}{dt}=k_1\cdot n(t)+k_2\cdot n^2+k_3\cdot n^3 \qquad (2)$$

The absorption intensity $\Delta A(t)$ of the ground-state blenching peak indicates the excited carrier density $n(t)$, and the linear relationship between $n(t)$ and $\Delta A(t)$ can be described as equation S1:

$$n(t)=C\cdot\Delta A(t) \qquad \text{(S1)}$$

Where C is the conversion coefficient between excited carriers and the absorption intensity. Usually, the value of C could be extracted from the equation S2:

$$C=\frac{n_0}{\Delta A_0} \qquad \text{(S2)}$$

The n_0 and ΔA_0 are the initial carrier density and maximal absorption intensity after pulsed excitation, respectively. Finally, we got C from the average of $n_0/\Delta A_0$ at three excitation powers P within the non-saturated absorption region (in Supplementary Fig. 10a and Supplementary Fig. 10c, the ΔA_0 grows linearly with P). Assuming that all the photons absorbed are converted to excited carriers, the n_0 could be estimated through the equation S3 below:

$$n_0=\frac{P}{f\cdot E_p d\cdot\pi\cdot r^2} \qquad \text{(S3)}$$

Where P is the energy power absorbed by perovskite films, f is the laser repetition rate, E_p is the photon energy of the pump laser, d is the film thickness, and r is the radius of the laser spot. Put the values of C into equation S1, we could obtain the carrier density $n(t)$. Taking the time derivative of $n(t)$, the curves of $-\frac{dn(t)}{dt}$ verse $n(t)$ is plotted in Fig. 2e and Fig. 2f. Then k_2 and k_3 could be extracted through the curve global fitting using equation 2[14,15].

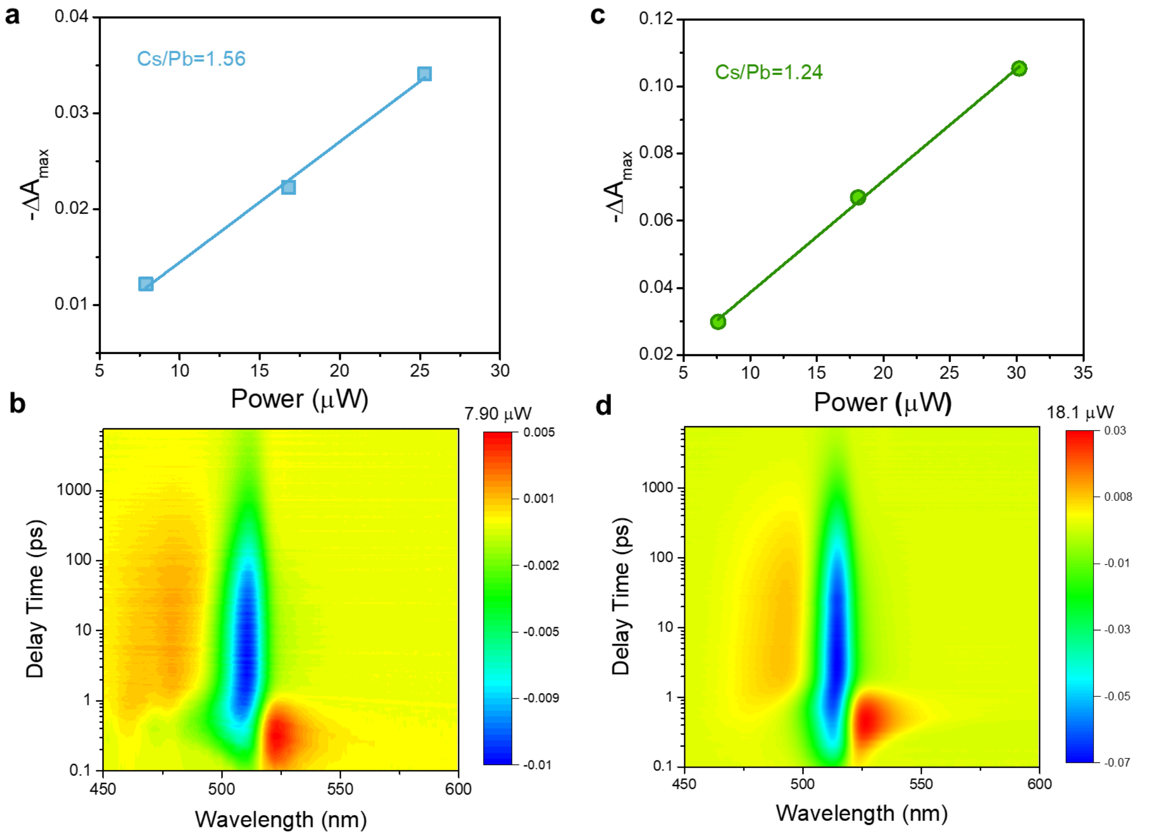

Supplementary Fig. 10 Transient absorption spectra. **a**, **c** Initial TA bleach signal plotted over pump power for Cs/Pb=1.56 and Cs/Pb=1.24, which indicate they are in the unsaturated absorption region. **b**, **d** The pseudo-color maps of Cs/Pb=1.56 and Cs/Pb=1.24 under the excitation power of 7.9 μW and 18.1 μW.

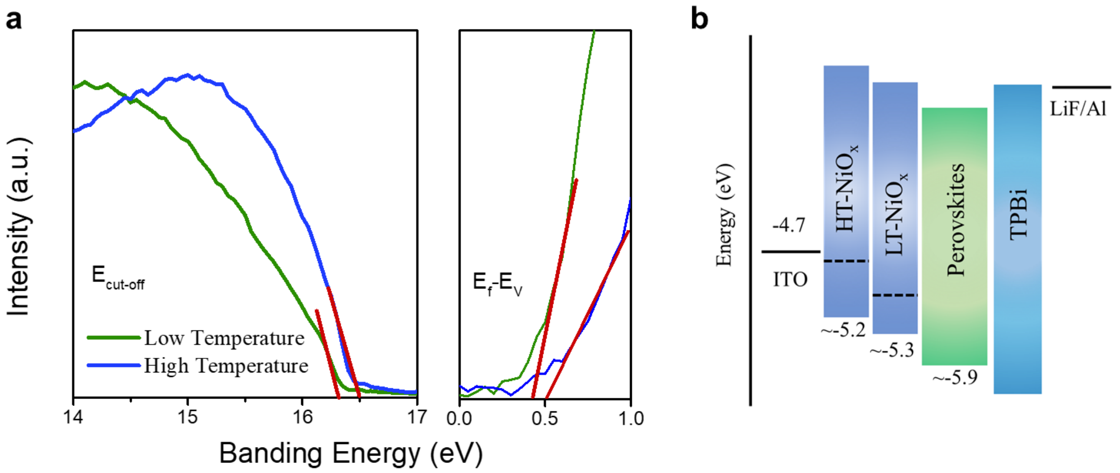

Supplementary Fig. 11 Ultraviolet Photoelectron Spectroscopy (UPS) spectra of NiO_x sputtered at low temperature (LT-NiO_x) and high temperature (HT-NiO_x). **a** The Fermi level (E_f=$E_{cut\text{-}off}$-21.22) and the valence band (E_f - $E_v \approx$ 0.4 eV for LT-NiO_x and 0.5 eV for HT-NiO_x). **b** The lower barrier between NiO_x and perovskites for LT-NiO_x is believed to promote the hole injection.

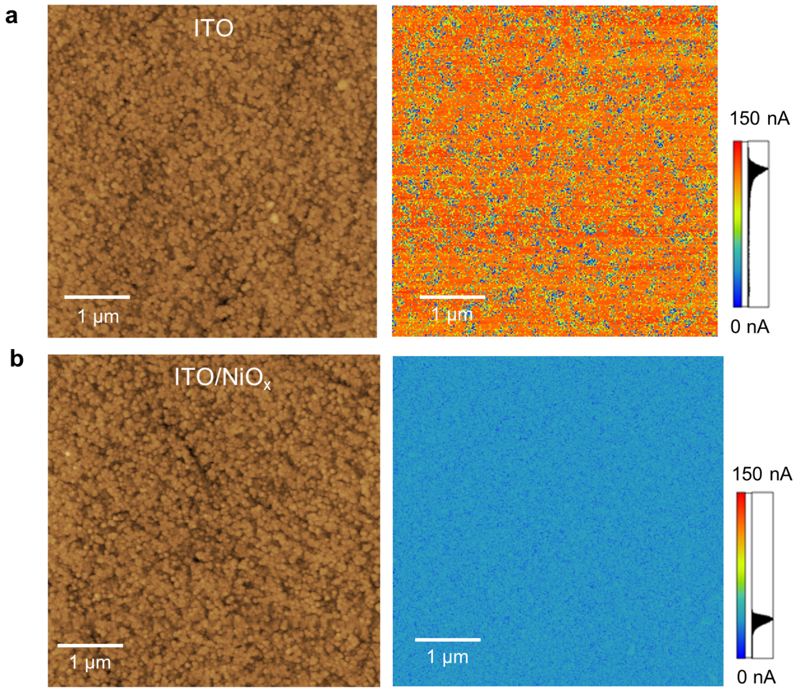

Supplementary Fig. 12 The conductive atomic force microscope (c-AFM) characterization of the sputtered NiO_x layer. **a** The c-AFM of ITO film. **b** The c-AFM of ITO/ NiO_x layer. The ITO/NiO_x film shows uniform conductivity without significant leakage, suggesting the good quality of the sputtered NiO_x.

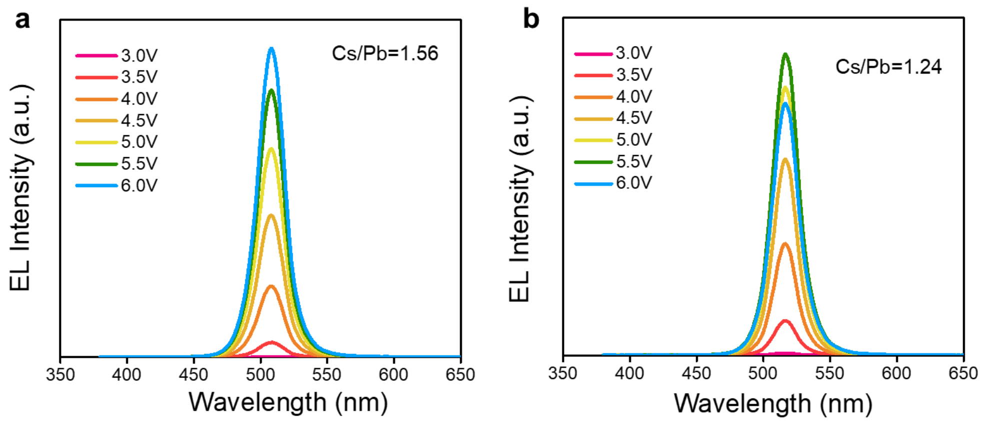

Supplementary Fig. 13 EL spectra of Cs/Pb=1.56 (a) and Cs/Pb=1.24 (b) at different voltage bias. As bias voltage increased from 3.0 to 6.0 V, the EL peak positions remain unchanged.

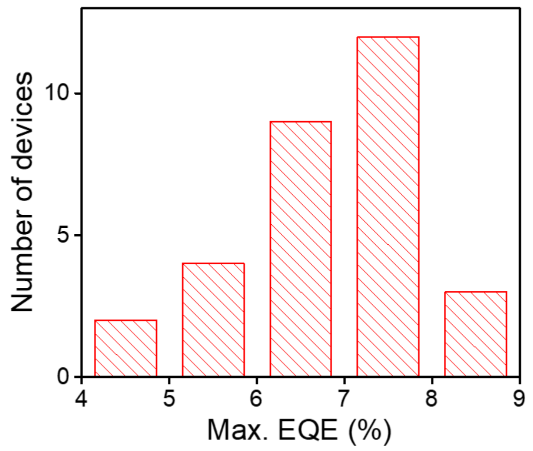

Supplementary Fig. 14 Histogram of max. EQE of 30 devices.

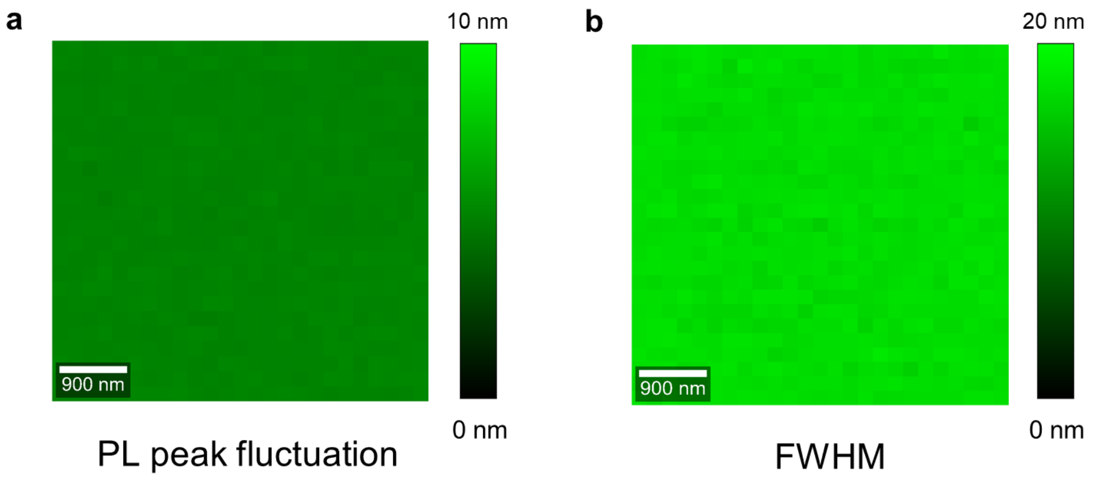

Supplementary Fig. 15 The spatial distribution maps of PL peak fluctuation (a) and FWHM (b) indicating outstanding uniformity of thermally evaporated perovskite film in the micro area. Noted that the scale bar in (a) is leveled.

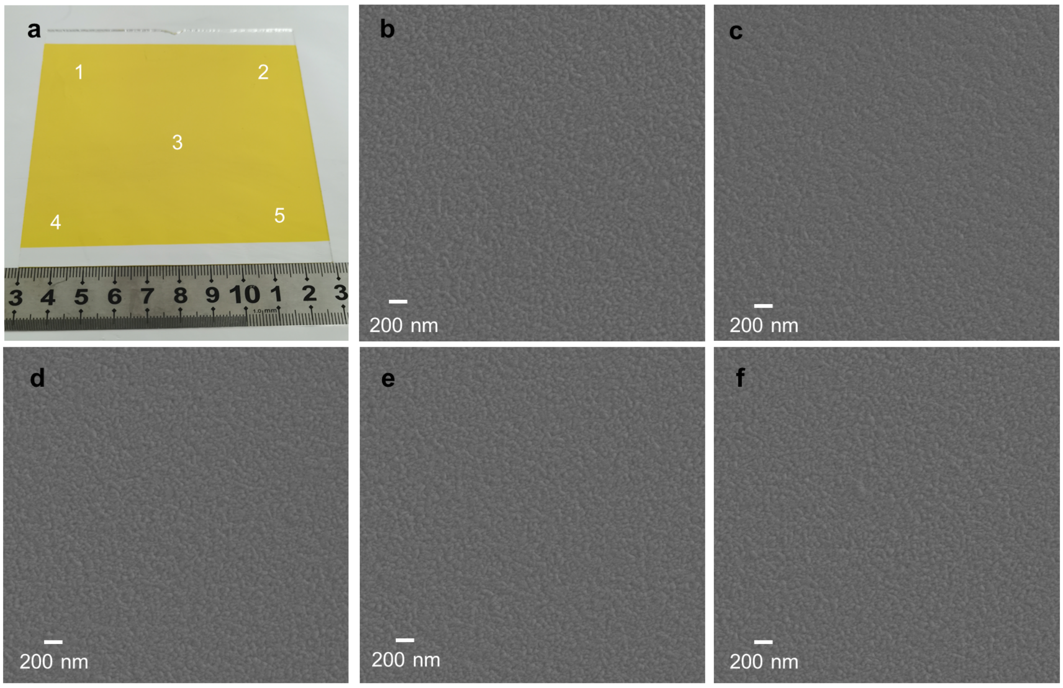

Supplementary Fig. 16 Morphology characterization of the large-area perovskite film. **a** The measured morphology of five regions covering the center and edge in the film. **b-f** Top-view SEM images (low magnification) of the spot 1-5, which are almost the same.

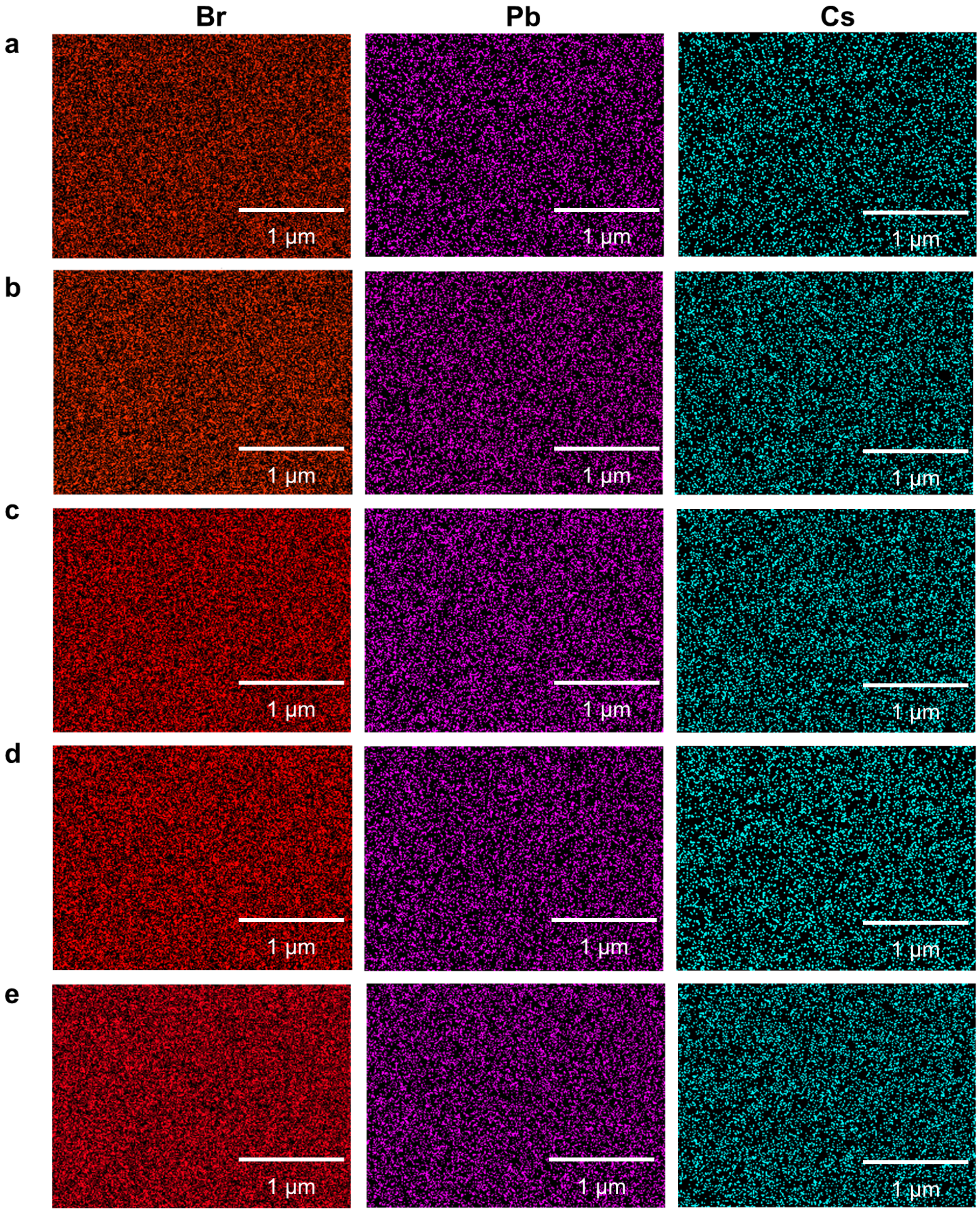

Supplementary Fig. 17 EDS mapping of the large-area perovskite film. **a-e** Cs, Pb, and Br mapping, which corresponding to the 1-5 regions illustrated in Supplementary Fig. 16a.

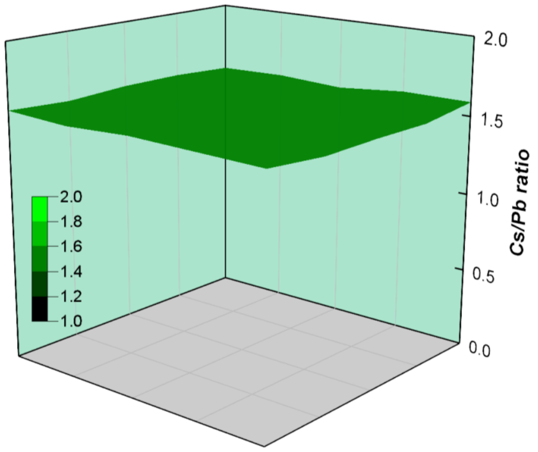

Supplementary Fig. 18 Composition characterization of the large-area perovskite film. We extracted the Cs/Pb ratios of EDS from 25 spots evenly distributed in the large-area film, and the fluctuations are negligible.

Supplementary Table 4 The reported large-area PeLEDs

Active layer	Emission region	Fabrication process	Area	Ref.
$FAPbI_3$	near-infrared	Spin-coating	900 mm^2	[16]
$FAPbI_3$	near-infrared	Spin-coating	120 mm^2	[17]
$FAPbBr_3$	Green	QD	400 mm^2	[18]
$MAPbI_3$	near-infrared	blade-coating	2800 mm^2	[7]
$CsPbBr_3$	Green	Thermal evaporation	1400 mm^2	[19]
**$CsPbBr_3$**	**Green**	**Thermal evaporation**	**4000 mm^2**	**This work**

Supplementary Note 2

The thermal evaporated Cs-Pb-Br films show fascinating morphology with dense grains over the substrate[20], which is a prerequisite for highly efficient LEDs. As shown in Supplementary Fig. S19, the Cs/Pb ratio will affect the morphology of thermally evaporated films, and the average grain size decreases from 93(±24) nm to 42(±10) nm as the Cs/Pb ratio increases from 1.24 to 1.77. The reason for that is because Cs_4PbBr_6 with zero-dimensional (0D) structure impede the growth of the $CsPbBr_3$ crystals[21-23]. Thereby, excess Cs_4PbBr_6 may lead to lower crystallinity of our $CsPbBr_3/Cs_4PbBr_6$ films (Supplementary Fig. S19c), and inadequate Cs_4PbBr_6 will enlarge grain sizes and induce rough morphology (Supplementary Fig. S19a). A moderate Cs/Pb ratio of 1.56 possesses a uniform and dense morphology (Supplementary Fig. S19b), laying the foundation for large-area and efficient PeLED fabrications.

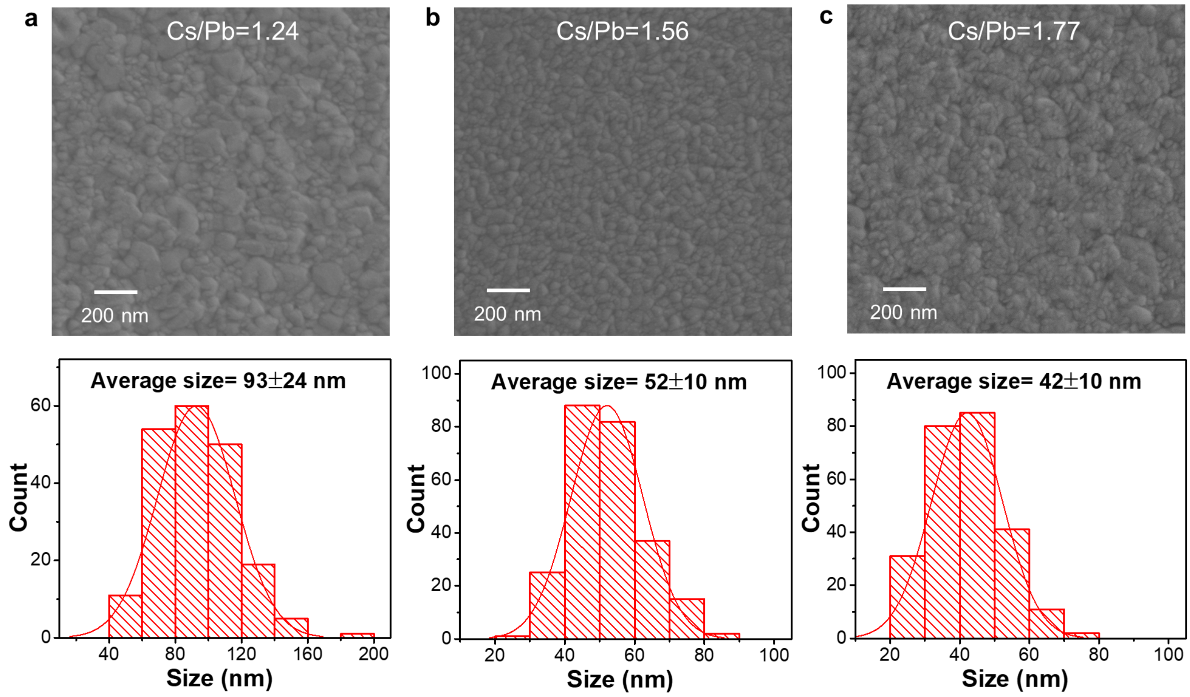

Supplementary Fig. 19 The top-view SEM of Cs-Pb-Br films and their corresponding grain size distributions are presented below. The films exhibit excellent compactness. From a to c, the Cs/Pb ratio is increased from 1.24 to 1.77. The scale bar is 200 nm.

Supplementary References

1. Cao, Y. et al. Perovskite light-emitting diodes based on spontaneously formed submicrometre-scale structures. *Nature* **562**, 249–253 (2018).
2. Lin, K. et al. Perovskite light-emitting diodes with external quantum efficiency exceeding 20 per cent. *Nature* **562**, 245–248 (2018).
3. Li, H. et al. Coffee ring elimination and crystalline control of electrohydrodynamically printed high-viscosity perovskites. *J. Mater. Chem. C* **7**, 14867-14873 (2019).
4. Yang, J. et al. Toward Full-Color Electroluminescent Quantum Dot Displays. *Nano Lett.* **21**, 26-33 (2021).
5. Li, D. et al. Inkjet Printing Matrix Perovskite Quantum Dot Light-Emitting Devices. *Adv. Mater. Technol.* **5**, 2000099 (2020).
6. Zhu, M. et al. Electrohydrodynamically Printed High-Resolution Full-Color Hybrid Perovskites. *Adv. Func. Mater.* **29**, 1903294 (2019).
7. Chu, S. et al. Large-area and efficient perovskite light-emitting diodes via low-temperature blade-coating. *Nat. Commun.* **12**, 147 (2021).
8. Liu, C., Cheng, Y.-B. & Ge, Z. Understanding of perovskite crystal growth and film formation in scalable deposition processes. *Chem. Soc. Rev.* **49**, 1653-1687 (2020).
9. Liu, X.-K. et al. Metal halide perovskites for light-emitting diodes. *Nat. Mater.* **20**, 10-21 (2021).
10. Wu, L. et al. From Nonluminescent Cs_4PbX_6 (X = Cl, Br, I) Nanocrystals to Highly Luminescent $CsPbX_3$ Nanocrystals: Water-Triggered Transformation through a CsX-Stripping Mechanism. *Nano Lett.* **17**, 5799-5804 (2017).
11. Shin, M. et al. Modulation of growth kinetics of vacuum-deposited $CsPbBr_3$ films for efficient light-emitting diodes. *ACS Appl. Mater. Interfaces* **12**, 1944–1952 (2020).
12. Xing, G. et al. Transcending the slow bimolecular recombination in lead-halide perovskites for electroluminescence. *Nat. Commun.* **8**, 14558 (2017).
13. Dong, Y. et al. Bipolar-shell resurfacing for blue LEDs based on strongly confined perovskite quantum dots. *Nat. Nanotechnol.* **15**, 668-674 (2020).
14. Richter, J. M. et al. Enhancing photoluminescence yields in lead halide perovskites by photon recycling and light out-coupling. *Nat. Commun.* **7**, 13941 (2016).
15. Chen, Z. et al. Recombination Dynamics Study on Nanostructured Perovskite Light-Emitting Devices. *Adv. Mater.* **30**, 1801370 (2018).
16. Zhao, X. & Tan, Z.-K. Large-area near-infrared perovskite light-emitting diodes. *Nat. Photonics* **14**, 215–218 (2020).
17. Xie, C., Zhao, X., Ong, E. W. Y. & Tan, Z.-K. Transparent near-infrared perovskite light-emitting diodes. *Nat. Commun.* **11**, 4213 (2020).
18. Wang, H. et al. A Multi-functional Molecular Modifier Enabling Efficient Large-Area Perovskite Light-Emitting Diodes. *Joule* **4**, 1977-1987 (2020).
19. Chen, C. et al. Efficient Flexible Inorganic Perovskite Light-Emitting Diodes Fabricated with $CsPbBr_3$ Emitters Prepared via Low-Temperature in Situ Dynamic Thermal Crystallization. *Nano Lett.* **20**, 4673-4680 (2020).
20. Ling, Y. et al. Enhanced Optical and Electrical Properties of Polymer-Assisted All-Inorganic Perovskites for Light-Emitting Diodes. *Adv. Mater.* **28**, 8983-8989 (2016).
21. Quan, L. N. et al. Highly Emissive Green Perovskite Nanocrystals in a Solid State Crystalline Matrix. *Adv. Mater.* **29**, 1605945 (2017).
22. Tong, G. et al. Phase transition induced recrystallization and low surface potential barrier leading to 10.91%-efficient $CsPbBr_3$ perovskite solar cells. *Nano Energy* **65**, 104015 (2019).
23. Li, H. et al. Interface engineering using a perovskite derivative phase for efficient and stable $CsPbBr_3$ solar cells. *Journal of Materials Chemistry A* **6**, 14255-14261 (2018).
